# Accuracy of D-dimer:fibrinogen ratio to diagnose pulmonary thromboembolism in patients admitted to intensive care units

**DOI:** 10.5830/CVJA-2012-041

**Published:** 2012-09

**Authors:** Shokoufeh Hajsadeghi, Scott R Kerman, Mojtaba Khojandi, Helen Vaferi, Roza Ramezani, Negar M Jourshari, Sayyed AJ Mousavi, Hamidezar Pouraliakbar

**Affiliations:** Department of Cardiology, Rasoul-e-Akram Hospital, Tehran University of Medical Sciences, Tehran, Iran; Department of Cardiology, Students Scientific Research Centre, Tehran University of Medical Sciences, Tehran, Iran; Department of Cardiology, Students Scientific Research Centre, Tehran University of Medical Sciences, Tehran, Iran; Department of Cardiology, Students Scientific Research Centre, Tehran University of Medical Sciences, Tehran, Iran; Department of Cardiology, Students Scientific Research Centre, Tehran University of Medical Sciences, Tehran, Iran; Department of Cardiology, Students Scientific Research Centre, Tehran University of Medical Sciences, Tehran, Iran; Department of Pulmonology, Rasoul-e-Akram Hospital, Tehran University of Medical Sciences, Tehran, Iran; Department of Radiology, Shahid Rajaee Heart Hospital, Tehran University of Medical Sciences, Tehran, Iran

**Keywords:** D-dimer, fibrinogen, pulmonary thromboembolism, intensive care unit (ICU)

## Abstract

**Introduction:**

Pulmonary thromboembolism (PTE) may increase D-dimer and decrease fibrinogen levels. However, in settings such as intensive care units (ICU) and in long-term hospitalised patients, several factors may influence D-dimer and fibrinogen concentrations and make them unreliable indicators for the diagnosis of PTE. The aim of this study was to evaluate the accuracy of D-dimer:fibrinogen ratio (DDFR) for the diagnosis of PTE in ICU patients.

**Methods:**

ICU patients who were suspected of having a first PTE and had no history of using anti-coagulants and contraceptives were included in the study. Levels of D-dimer and fibrinogen were measured for each patient prior to any intervention. Angiography or CT angiography was done in order to establish a definite diagnosis for each patient. Suitable analytical tests were performed to compare means.

**Results:**

Eighty-one patients were included in the study, of whom 41 had PTE and 40 did not. Mean values of D-dimer and fibrinogen were 3.97 ± 3.22 μg/ml and 560.6 ± 197.3 mg/dl, respectively. Significantly higher levels of D-dimer (4.65 ± 3.46 vs 2.25 ± 2.55 μg/ml, *p* = 0.006) and DDFR (0.913 ± 0.716 vs 483 ± 0.440 × 10^-3^, *p* = 0.003) were seen in PTE patients than in those without PTE. Receiver operating characteristic (ROC) analysis showed a 70.3% sensitivity and 70.1% specificity with a D-dimer value of 2.43 μg/ml (AUC = 0.714, *p* = 0.002) as the best cut-off point; and a 70.3% sensitivity and 61.6% specificity with a DDFR value of 0.417 × 10^-3^ (AUC = 0.710, *p* = 0.004) as the best cut-off point. In backward stepwise regression analysis, DDRF (OR = 0.72, *p* = 0.025), gender (OR = 0.76, *p* = 0.049) and white blood cell count (OR = 1.11, *p* = 0.373) were modelled (*p* = 0.029, R^2^ = 0.577).

**Conclusion:**

For diagnosis of PTE, DDFR can be considered to have almost the same importance as D-dimer level. Moreover, it was possible to rule out PTE with only a D-dimer cut-off value < 0.43 mg/dl, without the use of DDFR. However, these values cannot be used as a replacement for angiography or CT angiography

## Abstract

Pulmonary thromboembolism (PTE) is the third most common cause of cardiovascular-related deaths, with an average incidence of one in 100 000 patients annually.^1^,^2^ PTE is also one of the most important causes of sudden death and occurs in 10% of hospitalised patients, of which only 29% are correctly diagnosed before death.^3^ Moreover, PTE is a common life-threatening complication in patients with long-term hospitalisation, especially in intensive care units (ICU).^4^

The signs and symptoms of PTE are often very non-specific and can lead the practitioner to misdiagnose it.^5^ Although computed tomographic (CT) angiography is a first-line method for the diagnosis of PTE, it is contra-indicated in patients with renal insufficiency and in pregnant women, and it is relatively expensive, especially for developing countries. These limitations can result in mismanagement of PTE.^5^ Therefore attempts have been made for years to find a less-invasive, well-priced and more available test, such as biochemical markers in plasma.^7^-^10^

D-dimer is a degradation product of cross-linked fibrin that increases in acute thromboembolic events.^11^ D-dimer concentrations can be used to diagnose or rule out PTE but its specificity is poor because D-dimer levels can be elevated in other clinical conditions associated with additional fibrin formation, including old age, malignancies, infections and postoperative states.^12^,^13^

Plasma fibrinogen is one of the most important factors in the coagulation cascade and its concentration rises in many conditions, such as haemodynamic impairment, infections, cardiac, lung and aortic diseases and malignancies, as an acute-phase reactant. Many of these conditions have signs and symptoms similar to those of PTE.^14^,^15^

A study by Kucher *et al.* demonstrated that the D-dimer:fibrinogen ratio could be a specific predictor for PTE in emergency patients with no other medical condition.^9^ However, in other settings such as the ICU or in long-term hospitalised patients with an elevated risk for PTE, several factors may influence D-dimer and fibrinogen concentrations.^12^-^16^ Furthermore, in these patients, there was less accessibility to CT angiography and more complications were experienced with the use of it.^17^,^18^ The aim of this study was to evaluate the reliability of the D-dimer:fibrinogen ratio (DDFR) for the diagnosis of PTE in ICU patients.

## Methods

In this analytical cross-sectional study, 91 critically ill patients admitted to the ICU wards of Rasoul-e-Akram and Shahid-Rajaee hospitals were included. All of the patients were diagnosed with diseases such as heart failure, pneumonia and stroke at the time of hospitalisation. To enrol these patients in our study, they had to be susceptible to a first PTE in the ICU setting, and showing signs and symptoms of PTE.

Diagnosis was established by angiography or CT angiography. The patients with documented PTE were included in our case group and those without PTE were used as the control group. Patients with a history of using anticoagulants or oral contraceptives, and those with a previous history of PTE were excluded.

Prior to any treatment or invasive diagnostic studies, blood samples were taken from all patients for routine laboratory tests such as a complete blood cell count (CBC), arterial blood gas (ABG), and plasma sodium (Na), potassium (K), D-dimer and fibrinogen levels. Medical history and other demographic information were collected from patients’ medical files and inserted into pre-prepared checklists.

For D-dimer and fibrinogen assays, 2.7 ml of blood was taken from the antecubital vein of all patients, placed in standard Vacutainer (Becton Dickinson, Plymouth, UK) tubes containing 0.109 M buffered tri-sodium citrate, and centrifuged at 1 000 × *g* for 10 minutes at 18–21°C to extract the plasma. The samples were then sent to the laboratory in a cold box.

All biochemical assays were carried out in the clinical laboratory of Day General Hospital, Tehran, Iran. Functional fibrinogen level was measured by the Clauss method.^19^ D-dimer level was measured with a Tina-quant D-dimer diagnostic kit (Roche, Mannheim, Germany) by particle-enhanced immunoturbidimetric assay with the aid of an automated chemical analysis system (model 704, Hitachi, Tokyo, Japan). Intra- and inter-assay coefficients of variance of this test were 6.6 and 1.1%, respectively. In order to calculate DDFR, the equation below was used: DDFR = $\frac{D-dimer\;(\mu g/ml)}{Fibrinogen\;(mg/dl)}\times 100$ The study was pre-evaluated and approved by the ethics committee of the Iran University of Medical Sciences. All patients or their next of kin were aware of their presence in the study and verbal or written consent was given. All patients participated anonymously and their personal information was kept confidential.

## Statistical analysis

All data were entered and analysed by SPSS for Windows version 16. Qualitative data were expressed as percentages and quantitative data as means ± SD. Before the analysis, all data of quantitative variables were tested for normal distribution using the Kolmogorov–Smirnov test. Statistical tests such as the Student’s *t*-test, Chi-square and Mann–Whitney *U*-test were used. For calculating the sensitivity and specificity of various cut-off points for D-dimer and DDFR levels in this study, a receiver operating characteristics (ROC) analysis and curve was conducted. In addition, regression analysis was performed to create a model to evaluate the risk factors as a predictive test. A *p*-value < 0.05 was considered statistically significant.

## Results

## Baseline characteristics

After excluding 10 patients who did not meet our inclusion criteria, 81 patients were included in this study; 38 males and 43 females. Mean age was 61.62 ± 17.40 years and the mean duration of hospitalisation was 16.78 ± 12.1 days. The most common cause of admission was cardiovascular disorders (23 patients, 28.3%), pulmonary disease (21 patients, 25.9%) and neurologic disorders (12 patients, 14.8%). Other causes such as kidney disease, gastrointestinal bleeding and complications after orthopaedic surgery were seen in the remaining cases (24 patients, 29.6%).

In their medical history, 27 patients (33.3%) had diabetes mellitus (DM), 27 (33.3%) had a history of previous cardiac events (myocardial infarction, unstable angina and other cardiac problems), 40 (49.5%) had hypertension (HTN), and 20 patients (24.7%) had a history of any kind of surgery in the past three months.

At the end of the study, 41 patients (50.61%) were diagnosed as definite PTE cases and 40 (49.39%) had a diagnosis other than PTE and were considered our control group. From the Chi-square test, a significant difference was seen between gender percentages in the PTE and non-PTE groups, as 11 of the 41 (26.8%) PTE-positive patients were males, compared to 23 of 40 (56%) in the PTE-negative group (*p* = 0.001). Other characteristics are shown in Table 1.

**Table 1.** Baseline Characteristics Of The Patients Included In The Study, Divided By Patients With And Without PTE

	*All patients (n = 81)*	*With PTE (n = 41)*	*Without PTE (n = 40)*	p
Age (years)	61.62 ± 17.40	60.41 ± 14.85	61.85 ± 20.20	0.867^#^
Duration of hospitalisation (days)	16.78 ± 12.10	14.95 ± 11.65	19.05 ± 12.24	0.154^#^
CRP (mg/l)	24.65 ± 16.64	21.42 ± 18.00	27.11 ± 14.22	0.921^#^
Temperature (°C)	37.21 ± 0.62	36.98 ± 0.39	37.46 ± 0.75	0.091^#^
Systolic BP* (mmHg)	136.21 ± 24.96	136.60 ± 23.80	136.81 ± 26.92	0.980^#^
Diastolic BP* (mmHg)	83.81 ± 15.01	82.40 ± 16.79	85.56 ± 12.70	0.538^#^
Heart rate (/min)	88.47 ± 17.21	91.06 ± 16.36	85.56 ± 18.19	0.361^#^
Respiratory rate (/min)	22.36 ± 6.72	22.94 ± 7.22	21.67 ± 6.24	0.595^#^
Sodium (mEq/l)	139.36 ± 6.78	141.00 ± 7.36	137.58 ± 5.88	0.201^#^
Potassium (mEq/l)	4.36 ± 0.54	4.21 ± 0.50	4.54 ± 0.55	0.211^$^
WBC (/mm^3^)	11.07 ± 4.26	3.95 ± 1.02	4.22 ± 1.09	0.073^#^
Haematocrit (%)	37.00 ± 7.81	36.28 ± 7.49	37.77 ± 6.18	0.565^#^
pH	7.07 ± 0.76	6.84 ± 0.96	7.39 ± 0.06	0.343^$^
PO_2_ (mmHg)	72.63 ±22.68	64.73 ± 22.50	79.41 ± 21.54	0.258^#^
PCO_2_ (mmHg)	45.38 ± 17.99	47.76 ± 20.69	43.34 ± 16.74	0.684^#^
HCO_3_ (mEq/l)	27.15 ± 10.37	27.05 ± 7.69	27.25 ± 13.33	0.975^#^

^#^From independent samples t-test (for normally distributed variables). ^$^From Mann–Whitney U-test (for non-normally distributed variables).

Mean C-reactive protein (CRP) level was 24.65 ± 16.64 mg/l and white blood cell count (WBC) was 11.11 ± 4.12 /mm^³^. As shown in Table 1, none of the other parameters had significant differences between patients with and without PTE.

## D-dimer, fibrinogen and DDFR

The mean values of D-dimer and fibrinogen levels were 3.99 ± 3.19 μg/ml and 571.4 ± 196.1 mg/dl, respectively. Mean DDFR was 0.712 ± 0.643 × 10^-3^. As shown in Table 2, D-dimer and DDFR were significantly different between the PTE and non-PTE groups, but the fibrinogen level did not differ significantly.

**Table 2.** Concentration Of D-Dimer, Fibrinogen And DDFR In Patients With And Without PTE; Bold *p*-Values Are Significant

	*D-dimer (μg/ml)*	*Fibrinogen (mg/dl)*	*DDFR^#^ (× 10^-3^)*
PTE positive	4.65 ± 3.46	536.73 ± 186.32	9.13 ± 7.16
PTE negative	2.25 ± 2.55	586.33 ± 211.06	4.83 ± 4.40
p*	0.006	**0.298**	**0.003**

*From independent samples *t*-test. ^#^D-dimer:fibrinogen ratio.

**Fig. 1.**
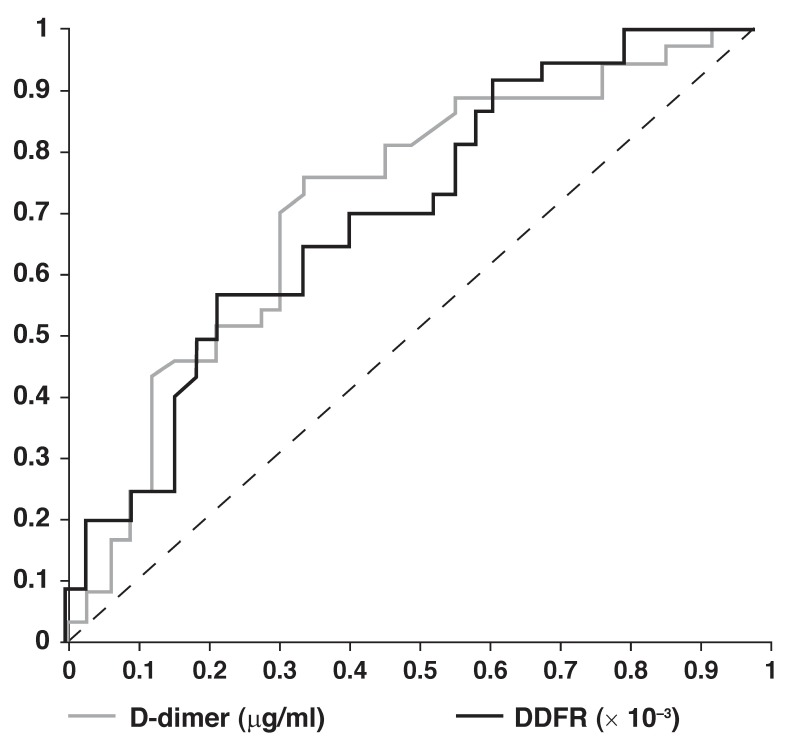
ROC curve for D-dimer and DDFR to diagnose PTE by different cut-off points; compare with Table 3..

In order to find the best cut-off points for D-dimer, fibrinogen and DDFR as diagnostic tests for PTE, a ROC analysis was performed. The ROC curve is used to calculate the area under the curve (AUC) as a measure of the diagnostic accuracy. Based on this analysis, for DDFR (AUC = 0.713, *p* = 0.003) a value of 0.105 × 10^-3^ was 100% sensitive and 1.621 × 10^-3^ was 100% specific for a diagnosis of PTE. Furthermore, this analysis on D-dimer values (AUC = 0.721, *p* = 0.002) showed a 100% sensitivity for 0.43 μg/ml and a 100% specificity for 11.5 μg/ml for the diagnosis of PTE. The same analysis on fibrinogen did not show any significant cut-off point (AUC = 0.410, *p* = 0.198) (Table 3, Fig. 1).

**Table 3.** Results From Roc Analysis; Bold Numbers Represent The Best Value Between All Cut-Off Points Calculated From The ROC Curve

		*Sensitivity (%)*	*Specificity (%)*	*Positive predictive value (%)*	*Negative predictive value (%)*	*Positive likelihood ratio*	*Negative likelihood ratio*	*Accuracy^$^ (%)*
D-dimer (μg/ml)	Best sensitivity (0.43)	**100**	6.9	54.1	100	1.07	-	55.7
	Best accuracy (2.43)	70.3	70.1	72.2	67.6	2.35	0.42	**70**
	Previously used# (7.0)	24.1	91.9	75	51.9	2.97	0.82	55.7
	Best specificity (11.5)	5.4	**100**	100	51.4	-	0.84	52.8
DDFR* (× 10^-3^)	Best sensitivity (.105)	**100**	22.2	58.73	100	1.2	-	62.8
	Best accuracy (.233)	91.9	40.4	62.9	81.2	1.54	0.2	**67.1**
	Previously used^#^ (1.0)	35.1	84.5	72.2	53.8	2.26	0.76	58.5
	Best specificity (1.32)	18.9	**100**	100	51.6	-	0.81	57.1

*D-dimer:fibrinogen ratio. ^#^The nearest cut-off points in our ROC analysis to the cut-off points used before in other articles and medical references. ^$^Calculated by: Accuracy (%) = $\frac{true\;positive+true\;negative}{total\;patients}\times 100$

Multiple logistic regression analysis was used to create a model to predict the risk for PTE using the significantly different independent variable studied at the level of *p* < 0.1. DDFR, gender, temperature and WBC were included in backward stepwise regression analysis. The first model chosen was the best statistically (*p* = 0.029, *R*^²^ = 0.577). The results are shown in Table 4.

**Table 4.** Results Of Logistic Regression Analysis Of Significantly Different Independent Variables At The Level Of < 0.1, Bold *p*-Value Is Considered Significant

	p	*Odds ratio*	*95% CI^$^*
DDFR (× 10^-3^)*	**0.025**	1.72	1.442–2.113
WBC (/mm^3^)^#^	0.373	1.11	0.875–1.454
Temperature	**0.001**	145	0.542–3.91 E3
Gender	**0.05**	0.76	0.003–2.12

*D-dimer:fibrinogen ratio. ^#^White blood cell count. ^$^95% confidence interval calculated.

## Discussion

No significant difference was found in arterial blood gas and complete blood count analysis between the hospitalised PTE and non-PTE patients. These results confirm other studies that showed arterial blood gas or its combination with other data could not be used to detect PTE, and if used alone, may lower the sensitivity and specificity.^20^,^21^

Fibrinogen (factor I) is a soluble plasma glycoprotein, synthesised by the liver and converted by thrombin into fibrin during blood coagulation.^22^ Fibrinogen may increase in acute and chronic conditions as an acute-phase reactant with the same signs and symptoms as PTE. There are few studies on the relationship between fibrinogen level and PTE. A study by Palla *et al.* showed that fibrinogen levels in PTE patients (498 ± 369 mg/dl) were similar to those without PTE (520 ± 268 mg/dl) (*p* = 0.29.23). These results are similar to our results, which demonstrated no significant difference between fibrinogen levels in the PTE and non-PTE patients. However, Kucher *et al.* showed that fibrinogen levels were significantly lower in patients with PTE (*p* < 0.0001).^9^

There is controversy about using fibrinogen levels as a reliable diagnostic test and it should not be used alone in order to diagnose PTE. In addition, the fibrinogen level is unpredictable. It can rise due to acute phases in the ICU or decrease in liver congestion due to right ventricular failure.

In recent years, several studies have been conducted to evaluate the accuracy of a diagnostic test to detect acute PTE in emergency settings. Most demonstrated that D-dimer tests with cut-off points near 0.5 μg/ml could be used as an exclusion test.^9^,^23^-^25^ As our study showed, D-dimer levels less than 0.43 μg/ml had a 100% sensitivity and a negative predictive value for ruling out PTE. This confirms data from various studies demonstrating the ability of D-dimer to rule out PTE.

The only study that evaluated the D-dimer:fibrinogen ratio in PTE was conducted by Kucher *et al.*^9^ They found that a ratio above 1.04 × 10^-3^ had 100% specificity and 57.6% sensitivity for PTE and a two-fold diagnostic rate compared to D-dimer alone, with a cut-off point of 7 μg/ml with 100% specificity and 29.4% sensitivity (57.6% vs 29.4%).^9^ A recent study however contradicted Kucher and co-workers’ results.

Calvo-Romero’s study did not reveal a lower fibrinogen level in PTE patients with a positive D-dimer level, although it showed the D-dimer test to be less sensitive (semi-quantitative latex agglutination D-dimer assay with 78% sensitivity). It also demonstrated that patients with PTE had fibrinogen levels within the normal range (200–400 mg/dl). However, the sample size of the study was small compared to other studies in this field (40 cases).^26^

The aim of our study was to determine whether there was an inverse relationship between D-dimer and fibrinogen levels. The theory was that while the activation of the coagulation cascade consumes fibrinogen in the pulmonary vasculature to form fibrin, the activation of fibrinolysis results in elevated fibrin degradation products such as D-dimer.^9^ This theory may be applicable in acute PTE without complications and for any other factor that may influence D-dimer and fibrinogen (as an acute-phase reactant) concentrations in out-patients. In patients with other complications, these biomarkers will be different.^11^-^15^

We hypothesised that the conditions influencing D-dimer and fibrinogen levels would magnify the difference between these biomarkers when combined, and therefore lead to a more accurate diagnosis. As our study shows, when using the same cut-off points that Kucher *et al*. presented, D-dimer > 7 μg/ml was 24% sensitive and 91.9% specific, and DDFR > 10^-3^ was 35.1% sensitive and 84.5% specific. Based on our study, D-dimer > 2.43 μg/ml and DDFR > 0.233 × 10^-3^ had the best accuracy (70 and 67.1%, respectively). However neither was accurate enough to be used alone for the diagnosis of PTE in the ICU setting or in long-term hospitalised patients suspected of having PTE.

## Study limitations

Up to the end of the first phase of our study, 91 patients were enrolled and after filtering by the exclusion criteria, 81 patients were included. Due to the use of antithrombotic agents and good medical care, the incidence of PTE was low in the two hospitals where we collected the samples. Therefore we could not divide the patients into groups with different setting, such as medical ICU and surgical ICU, in order to evaluate the influence of different settings on D-dimer level and DDFR. We recommend a study to compare fibrinogen and D-dimer levels and DDFR in different settings and also in emergency departments as a unique study to make the comparison more reliable.

## Conclusion

No significant difference was found in the biochemical assays between the hospitalised PTE and non-PTE patients. Moreover, the significant difference in DDFR originated from D-dimer and not fibrinogen levels. Therefore DDFR appears to be almost as useful as D-dimer in diagnosing PTE in the ICU setting. In addition, it was possible to rule out PTE with only the D-dimer cut-off value of < 0.43 μg/ml without using DDFR. However, neither of these evaluations could replace angiography or CT angiography.
